# Rare variant analysis in multiply affected families, association studies and functional analysis suggest a role for the *ITGΒ4* gene in schizophrenia and bipolar disorder

**DOI:** 10.1016/j.schres.2018.03.001

**Published:** 2018-09

**Authors:** N.L. O'Brien, A. Fiorentino, D. Curtis, C. Rayner, C. Petrosellini, M. Al Eissa, N.J. Bass, A. McQuillin, S.I. Sharp

**Affiliations:** a UCL Molecular Psychiatry Laboratory, Division of Psychiatry, University College London, London, UK; b UCL Genetics Institute, University College London, London, UK; c Centre for Psychiatry, Barts and the London School of Medicine and Dentistry, London, UK

**Keywords:** Sequencing, Genotyping, Psychosis, Genetic risk

## Abstract

Recent results imply that rare variants contribute to the risk of schizophrenia. Exome sequence data from the UK10K project was used to identify three rare, amino acid changing variants in the *ITGB4* gene which segregated with schizophrenia in two families: rs750367954, rs147480547 and rs145976111. Association analysis was carried out in the exome-sequenced Swedish schizophrenia study and in UCL schizophrenia and bipolar cases and controls genotyped for these variants. A gene-wise weighted burden test was performed on a trio sample of schizophrenia cases and their parents. rs750367954 was seen in two Swedish cases and in no controls. The other two variants were commoner in cases than controls in both Swedish and UCL cohort samples and an overall burden test was significant at p = 0.0000031. The variants were not observed in the trio sample but *ITGB4* was most highly ranked out of 14,960 autosomal genes in a gene-wise weighted burden test. The effect of rs147480547 and rs145976111 was studied in human neuroblastoma SH-SY5Y cells. Cells transfected with both variants had increased proliferation at both 24 and 48 h (p = 0.013 and p = 0.05 respectively) compared to those with wild-type *ITGB4*. Taken together, these results suggest that rare variants in *ITGB4* which affect function may contribute to the aetiology of schizophrenia and bipolar disorder.

## 1 Introduction

The results of exome sequence studies imply a role for rare variants in the aetiology of schizophrenia (SCZ). Most studies to date have implicated pathways and sets of genes rather than individual genes (Curtis and UK10K Consortium, 2016; Genovese et al., 2016; Purcell et al., 2014; Singh et al., 2017), however, a combined analysis of case control and trio sequence data has implicated rare loss of function variants in the *SETD1A* gene (p = 3.3 × 10^−9^) in the aetiology of SCZ (Singh et al., 2016).

Analysis of sequencing data from multiply affected extended pedigrees can be valuable for identifying extremely rare variants that are transmitted to affected individuals (Curtis, 2011) and this approach has been successful in identifying variants in the *PLD3* (phospholipase D3) gene in late-onset Alzheimer's disease (Cruchaga et al., 2014). Recently a whole genome-sequencing study identified two different rare, protein-truncating variants in the *RBM12* gene cosegregating with psychosis in two different pedigrees (Steinberg et al., 2017). Here we report the discovery and follow-up of rare, protein-changing variants which were observed to cosegegrate with SCZ in subjects from two multiply-affected families which were sequenced as part of the UK10K project (Muddyman et al., 2013; UK10K Consortium et al., 2015).

## 2 Material and methods

### 2.1 Subjects

Four independent datasets were used. These comprised:

#### 2.1.1 UK10K cohort

We used British subjects from the UK10K dataset comprising 1392 with SCZ and 982 with severe childhood obesity (SCOOP) not known to have mental illness which have been described elsewhere (Curtis and UK10K Consortium, 2016). UK10K subjects were exome sequenced with coverage of 72× as described in full elsewhere (UK10K Consortium et al., 2015). Among the UK10K SCZ subjects were 48 subjects from 16 published and unpublished families multiply affected with SCZ that had been collected at UCL (Kalsi et al., 1994; Sherrington et al., 1988). Exome data was available for between 2 and 5 affected members per pedigree.

#### 2.1.2 Swedish SCZ study

The Swedish SCZ study consisted of the 2545 controls and 2545 cases with SCZ from Sweden for whom whole exome sequence data is available *via* dbGaP (Purcell et al., 2014). Detailed sample descriptions have been provided previously (Purcell et al., 2014).

#### 2.1.3 Bulgarian trio sample

The Bulgarian trio sample consisted of probands with SCZ and their parents (Fromer et al., 2014; Rees et al., 2015). The sample comprised whole exome sequence data from 591 trios, consisting of probands with SCZ and their parents, five of whom were also affected (Fromer et al., 2014; Rees et al., 2015). The short read files were downloaded from dbGaP along with family structure and phenotype information.

#### 2.1.4 UCL case-control samples

The UCL case-control samples sample and the recruitment methods have been previously described (Al Eissa et al., 2017; Fiorentino et al., 2014). Briefly, the sample comprised 1917 bipolar disorder (BP) participants, 1304 SCZ participants and 1348 control participants recruited from the UK.

### 2.2 Variant selection and bioinformatic prediction of variant impact

Custom-written software was used to annotate and identify rare, possibly functional variants shared between affected pedigrees members in the UCL samples included in UK10K. The impact of these variants was predicted using the PolyPhen-2 (Adzhubei et al., 2013) and Sort Intolerant from Tolerant (SIFT)(Kumar et al., 2009) bioinformatics tools.

### 2.3 Sanger sequencing in families F047 and F158

**Fig. 1:**
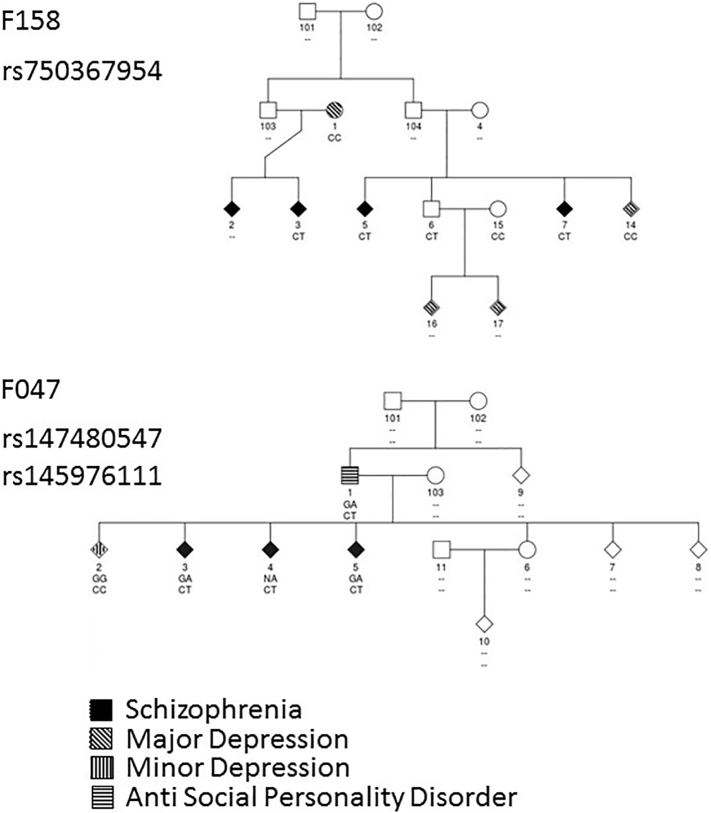
Non-synonymous *ITGΒ4* variants in two families multiply affected by schizophrenia and other psychopathology. unk Genotype not determined; filled shapes represent individuals suffering from schizophrenia; shaded shapes represent other psychiatric diagnoses as indicated.

Exome sequence data was available for three members of family F158 (subjects 3, 5 and 7) and for two members of family F047 (subjects 3 and 5; Fig. 1) from the UK10K sample. Transmission of rs750367954 (allele T) in family F158 and of rs147480547 (allele A) and rs145976111 (allele T) in family F047 was sought in all additional family members with available DNA by PCR and Sanger sequencing (Fig. 1). There was a limited amount of DNA available for individual 4 from family F047 and it was not possible to verify the genotype status of rs147480547 in this individual (Fig. 1).

### 2.4 Variant selection and genotyping

Non-synonymous variants segregating with SCZ in families F047 and F158 were selected for genotyping in the SCZ, BP and control samples. Genotyping was performed in-house with allele-specific PCR using KASPar reagents (LGC Genomics, Hoddesdon, UK) on a LightCycler 480 (Roche, Burgess Hill, UK) real-time PCR machine. Allele-specific primers were designed for each of the SNPs using Primer Picker (LGC Genomics) as shown in Supplementary Table 1. Genotyping of each heterozygote sample was repeated at least twice and known heterozygote individuals were included on each genotyping plate to ensure reliable calling of heterozygote subjects.

### 2.5 Variant calling in trios

The short read files from the Bulgarian trio sample were converted to fastq files using the fastq-dump utility of the dbGaP SRA toolkit. Reads were then aligned to the hg19 human reference sequence (build GRCh37) using Novoalign V3.02.08 (NovoCraft Technologies), duplicate reads were marked using SAMBLASTER (Faust and Hall, 2014) and the BAM files were sorted using Novosort V1.03.09 (NovoCraft Technologies). Genotypes were called according to GATK best practices (Broad Institute). The HaplotypeCaller module of GATK V3.6 was used to produce gVCF files and these were then combined using the CombineGVCFs module. Initial calls were made using the GenotypeGVCFs module and then SNPs were filtered based on accuracy estimates produced by VariantRecalibrator and indels were filtered using the VariantFiltration module and the filtering expression “QD < 2.0 || FS > 50.0 || ReadPosRankSum < −20.0”.

### 2.6 Statistical analysis

#### 2.6.1 Case-control analysis

Allelic association analyses for the genotyped SNPs were performed using Fisher's exact test in the Swedish and UCL samples separately and in combination with each other. A burden test was performed by combining information from all genotyped SNPs to compare the total variant allele counts between cases and controls. Statistical analyses were performed using R (R Core Team, 2017).

#### 2.6.2 Weighted burden analysis of whole exome sequence data from schizophrenia trios

The exome-sequenced trios were used to perform a gene-wise weighted burden analysis across all autosomal genes. Genotypes of autosomal variants in each gene were exported to SCOREASSOC (Curtis, 2012; Curtis and UK10K Consortium, 2016), which was used to construct a sample of pseudo-controls with genotypes consisting of the untransmitted alleles from the parents of each affected proband. A number of QC measures were applied. Variants were excluded if they did not have a PASS in the information field and individual genotype calls were excluded if they had a genotype quality score < 60. Variants were also excluded if >10% of cases or of pseudo-controls failed this quality threshold or if the heterozygote count was smaller than both homozygote counts in both cohorts. Indel calls seemed to produce artefactual results so were excluded. SCOREASSOC was used to carry out gene-wise weighted burden tests as previously described (Curtis, 2012; Curtis and UK10K Consortium, 2016) on the 591 pairs of cases and pseudo-controls to test whether there was an excess of rare, functional variants among cases. Only variants with a frequency of <0.01 in cases or pseudo-controls were considered. Functional weights were assigned so that variant types deemed more likely to have a functional effect were assigned higher weights in an approach similar to that recently suggested for dealing with *de novo* mutations (Jiang et al., 2015). Stop gain mutations were allocated a weight of 20. Non-synonymous variants were assigned a weight of 10. Stop loss and splice site variants are assigned a weight of 5. Other variants were not considered. To carry out a weighted burden analysis a score was calculated for each subject consisting of the sum of the weights of the variant alleles carried by that subject. A gene-wise test for association was then performed using a *t*-test to see if the average scores for the cases were higher than for the pseudo-controls, indicating that the cases tend to have an excess of rare, functional variants. The result of this weighted burden test was summarised as a signed log p (SLP), which is the logarithm base 10 of the two-tailed p value, given a positive sign if the mean score is higher for cases than pseudo-controls.

### 2.7 Cell lines

Undifferentiated SH-SY5Y human neuroblastoma cell lines were obtained from Sigma Aldrich (Gillingham, UK). The cells were cultured as mono layers in DMEM (Dulbecco's Modified Eagle Media), 10% FBS (Fetal Bovine Serum), 1% Penicillin (50 U/ml)/Streptomycin (50 μg/ml) maintained at 37 °C and 5% CO_2_. Cells were not passaged beyond passage 25.

### 2.8 *ITGB4* plasmid constructs

A TrueORF gold *ITGB4* plasmid with a c-terminal Myc-DDK tag was obtained from Origene (RC220541, Origene Technologies, Rockville, MD, USA). The *ITGB4* genetic variants rs147480547 allele A and rs145976111 allele T were introduced into the plasmid using the Q5® Site-Directed Mutagenesis Kit (New England Biolabs) and following the manufacturer's protocol. A plasmid containing both of the genetic variants was also constructed using the Q5® Site-Directed Mutagenesis Kit (New England Biolabs, UK).

### 2.9 Cell transfections

SH-SY5Y cells were transfected using lipofectamine 2000 (Life technologies, UK). Growth media was removed and cells were washed with PBS. Growth media was then replaced with Opti-MEM® reduced media serum (Life technologies, UK). Cell samples were transfected with four different DNA plasmid constructs, wildtype *ITGB4*, *ITGB4* rs147480547 allele A, *ITGB4* rs145976111 allele T and a plasmid construct containing both variants in the *ITGB4* cDNA. Successful transfection was determined using quantitative RT-PCR (not shown).

### 2.10 Cell proliferation assay

Cells were seeded at a density of 1x10^5^cells/cm^2^ and incubated for 24 and 48 h after transfection before cell proliferation was assayed. MTT (3-(4, 5-dimethylthiazol-2-yl)-2, 5-diphenyl tetrazolium bromide) was used to measure cellular proliferation (Sigma Aldrich, Gillingham, UK)(Mosmann, 1983). MTT was dissolved in PBS at 5 mg/ml and filtered-sterilised and then added to cells at 24 h and 48 h. Cells were incubated at 37 °C for 3 h. After 3 h, the media was removed and 0.04 N HCL-isopropanol was added to solubilise the converted formazan. Absorbance of formazan was measured at a wavelength of 570 nm with a background absorbance of 630-690 nm. *t*-Tests were performed on the MTT data using R.

## 3 Results

### 3.1 Variant identification

Among the sixteen UCL pedigrees exome sequenced as part of the UK10K study, the only gene in which rare (observed at a frequency of <1% in the UK10K dataset), protein-changing variants co-segregated with SCZ in more than one pedigree was *ITGB4*. One variant, rs750367954 (C > T), had not been previously reported at the time of analysis and was found in three affected members, two siblings and one first cousin, from family F158 (Fig. 1). The variant was also found in another sibling from family F158 who was not known to have a mental disorder but no follow up of mental health status was possible in this individual. This variant was not found in any other UK10K subjects. Two previously reported rare variants were identified in family F047: rs147480547 (G > A) which was shared by three affected siblings and rs145976111 (C > T) which was shared by two of them but the third sibling had an unknown genotype (Fig. 1). The minor allele (A) of rs147480547 was present in two additional UK10K SCZ subjects (minor allele frequency (MAF) = 0.0007) while minor allele (T) of rs145976111 was present in 7 additional UK10K SCZ subjects (MAF = 0.0025) and 3 SCOOP subjects (MAF = 0.0015).

**Table 1:** Prediction of variant impact on protein structure from Poly-Phen2 and SIFT for isoform 1 of *ITGΒ4* (NCBI reference sequence, NM_000213).

Variant ID [nucleotide change]	Amino acid change	Poly-Phen2	SIFT	Likely function & domain
rs147480547 [G/A]	Ala/Thr (A808T)	Benign (0.006–0.335)	Deleterious (0.01)	Unknown/Undefined
rs145976111 [C/T]	Arg/Cys (R977C)	Probably damaging (0.95–0.99)	Deleterious (0)	CALX-BETA Domain
rs750367954 [C/T]	Ala/Val (A1689V)	Benign (0–0.011)	Deleterious (0.014)	Protein:protein interaction with ERBIN; fibronectin type-III & immunoglobulin-like fold domains

rs750367954 C > T leads to a non-conservative amino acid change from alanine to valine at position 1689 (A1689V) of the main isoform of *ITGΒ4* (NM_000213; Supplementary Fig. 1). The A1689V amino acid substitution was predicted to be “Benign” with a score between 0 and 0.011 depending on the isoform by PolyPhen-2 (Supplementary Table 2) and “Deleterious” with a score of 0.01 by SIFT (Table 1). A1689V is present in an experimentally demonstrated protein interaction domain for ERBIN (Favre et al., 2001) and in Fibronectin type III and Immunoglobulin-like fold domains as predicted by INTERPRO (https://www.ebi.ac.uk/interpro/protein/P16144; Supplementary Fig. 1). rs147480547 G > A causes a non-conservative amino acid change from alanine to threonine at position 808 (A808T) of NM_000213. The A808T amino acid substitution was predicted to be “Benign” with a score between 0.006 and 0.335 depending on the isoform by PolyPhen-2 and “Deleterious” with a score of 0.01 by SIFT. rs145976111 C > T causes a non-conservative amino acid change from arginine to cysteine at position 977 (R977C) of NM_000213 (Supplementary Fig. 1). The R977C amino acid substitution was predicted to be “Probably Damaging” with a score between 0.95 and 0.999 depending on the isoform by PolyPhen-2 (Supplementary Table 2) and “Deleterious” with a score of 0 by SIFT.

### 3.2 Association analysis

Genotype counts for the variants in the Swedish SCZ cases and controls and the UCL samples are shown in Table 2. Results for the UCL SCZ and BP cases are shown separately and combined. BP subjects were included in the genotyping panel to allow assessment of the frequency of variants across the psychosis spectrum. rs750367954 C > T was detected in two Swedish cases but no other subjects and the other variants were commoner in cases than controls in both the Swedish and UCL cohorts. The data shows evidence for association with schizophrenia and bipolar disorder separately (p = 0.0022 and p = 0.0011 respectively for rs145976111) and for both disorders when the data was combined (p = 0.0004 for rs145976111). Table 2 also shows the results obtained from comparing combined genotype counts for the Swedish and UCL cases with other subjects, both for individual variants and for a burden test combining counts across all variants. The burden test was significant at p = 0.0000031.

**Table 2:** Genotypes of *ITGΒ4* variants in the cases and controls from the Swedish schizophrenia cohort (SE); the UCL cohorts (UK); the two cohorts combined; and for a burden analysis using genotypes combined across all three variants. Significance values were obtained using Fisher's exact test. Variant positions are shown according to hg19 (GRCh37).

Variant ID (Position on Chr17) [Base pair change]	Sample	Genotype counts	MAF	P	Odds ratio
		AA/AB/BB			(95% CI)
rs750367954	SE SCZ	2534/2/0	0.00039	0.25	Inf (0.19-Inf)
(73753036)	SE control	2540/0/0	0		
[C/T]	UK SCZ	1275/0/0	0	NA	NA
	UK BP	1893/0/0	0	NA	NA
	UK SCZ&BP	3168/0/0	0	NA	NA
	UK control	1324/0/0	0		0
	Combined SCZ	3802/2/0	0.0002	0.25	Inf (0.19-Inf)
	Combined BP	1892/0/0	0	NA	NA
	Combined BP&SCZ	5694/2/0	0.00018	0.52	Inf (0.13-Inf)
	Combined control	3844/0/0	0		
rs147480547	SE SCZ	2532/4/0	0.00079	0.062	Inf (0.66-Inf)
(73736128)	SE control	2540/0/0	0		
[G/A]	UK SCZ	1257/4/0	0.0016	0.21	4.16 (0.41–204.92)
	UK BP	1885/6/0	0.0016	0.25	4.16 (0.50–191.48)
	UK BP&SCZ	3142/10/0	0.0016	0.19	4.16 (0.59–180.66)
	UK control	1310/1/0	0.0004		
	Combined SCZ	3789/8/0	0.0011	0.021	8.12 (1.09–359.83)
	Combined BP	1885/6/0	0.0016	0.0064	12.24 (1.48–561.34)
	Combined BP&SCZ	5674/14/0	0.0012	0.0069	9.49 (1.44–400.60)
	Combined control	3850/1/0	0.00013		
rs145976111	SE SCZ	2522/12/0	0.0024	0.021	4.02 (1.08–22.19)
(73738809)	SE control	2537/3/0	0.00059		
[C/T]	UK SCZ	1258/10/0	0.0039	0.054	3.45 (0.89–19.53)
	UK BP	1876/14/0	0.0037	0.081	3.24 (0.90–17.60)
	UK SCZ&BP	3134/24/0	0.0038	0.035	3.32 (1.01–17.26)
	UK control	1306/3/0	0.0011		
	Combined SCZ	3780/22/0	0.0029	0.0022	3.72 (1.46–11.22)
	Combined BP	1876/14/0	0.0037	0.0011	4.76 (1.72–15.14)
	Combined BP&SCZ	5656/36/0	0.0032	0.0004	4.07 (1.70–11.81)
	Combined control	3843/6/0	0.00078		
Burden analysis	SCZ	11,371/32/0	0.0014	0.000038	
	BP	5653/20/0	0.0018	0.000015	
	BP&SCZ	17,024/52/0	0.0015	0.0000031	
	Control	11,537/7/0	0.00078		

### 3.3 Weighted burden analysis of trios

In the Bulgarian trio dataset no variants were observed at rs147480547 G > A, rs145976111 C > T or rs750367954 C > T. However, a gene-wise weighted burden test was carried out and provided informative results for 14,960 autosomal genes (Supplementary Table 3). A QQ plot showed that the SLPs obtained closely conformed to the null hypothesis distribution and no gene was exome-wide significant (Supplementary Fig. 2). However, out of all these genes *ITGΒ4* was most highly ranked with an SLP of 4.3, corresponding to a p value of 0.00005. Table 3 shows the genotype counts for *ITGΒ4* in cases and pseudo-controls. It can be seen that the result is driven by an excess of variants among cases at a variety of different positions (Supplementary Fig. 1).

**Table 3:** Genotype counts for rare variants in *ITGΒ4* in trio cases and pseudo-controls consisting of non-transmitted parental alleles. Using the weighted burden test implemented in SCOREASSOC, the excess in cases is significant at p = 0.00005. Amino acid changes are shown for NM_000213. NS Nonsynonymous.

Position on chr17 (hg19)	Variant identifier, base pair and amino acid change	Type	Pseudo-controls			Schizophrenia cases		
			AA	AB	BB	AA	AB	BB
73724461	rs775438066 G/A; R158Q	NS	589	0	0	586	3	0
73724543	17:73724543 G/A; M185I	NS	576	0	0	575	1	0
73726959	17:73726959 C/T; L336F	NS	585	0	0	584	1	0
73726989	rs778405226 G/C; G346R	NS	583	0	0	581	2	0
73727379	rs762334700 G/A; R382Q	NS	534	0	0	533	1	0
73728226	rs373717293 T/C;	Splice site	587	0	0	586	1	0
73728300	rs8079267 G/T; Q478H	NS	586	2	0	581	7	0
73729749	rs772615108 C/T; R545C	NS	546	1	0	547	0	0
73736505	rs760130077 G/A; R838Q	NS	542	0	0	541	1	0
73738731	rs764035400 C/T; R951W	NS	533	0	0	531	2	0
73746859	17:73746859 C/G; D1191E	NS	576	1	0	577	0	0
73746884	rs75129664 G/A; G1200R	NS	567	4	0	560	11	0
73750828	17:73750828 G/A; R1497H	NS	583	0	0	582	1	0
73752635	rs538467153 C/T; R1612C	NS	576	0	0	575	1	0
73753143	rs762581242 G/A; G1725R	NS	535	0	0	534	1	0
73753152	rs759302516 C/T; R1728C	NS	543	0	0	542	1	0
73753278	rs200010813 C/T;	Splice site	532	3	0	531	4	0
73753310	rs151327791 G/A; G1750R	NS	540	0	0	539	1	0
73753326	rs140577812 C/T; P1755L	NS	543	1	0	543	1	0
73753574	rs773923807 C/T; R1803W	NS	557	0	0	556	1	0

### 3.4 The impact of ITGB4 coding variants rs147480547 and rs145976111 on SH-SY5Y cell proliferation

Quantitative PCR analysis confirmed overexpression of *ITGΒ4* in SH-SY5Y cells transfected with the *ITGB4* plasmid constructs compared to SH-SY5Y cells transfected with an empty vector.

**Fig. 2:**
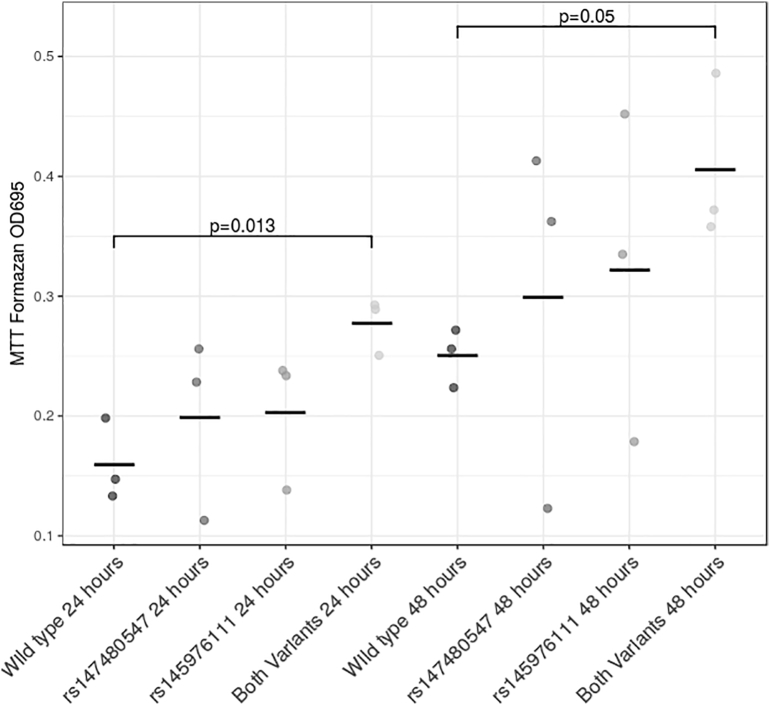
MTT-formazan formation from SH-SY5Y cells overexpressing the wildtype, rs147480547, rs145976111 and both variants of the *ITGΒ4* gene. The data was obtained from three independent experiments.

No gross morphological changes were observed in the cells transfected with the wildtype or variant ITGB4 plasmid constructs. Next we tested whether the variants would have an impact on SH-SY5Y cell proliferation on the basis that Integrin β4 is thought to play a role in neuronal survival and apoptosis signal transduction pathways (Lv et al., 2008; Lv et al., 2006; Su et al., 2007). Cells transfected with *ITGB4* constructs containing variant alleles for both rs147480547 and rs145976111 had increased MTT-formazan formation at both 24 and 48 h (p = 0.013 and p = 0.05 respectively) (Fig. 2). Transfection with *ITGB4* constructs expressing these variants separately produced intermediate values.

## 4 Discussion

One approach to detect rare variants is to utilise segregation information from multiple affected individuals within a family (Cruchaga et al., 2014; Curtis, 2011; Timms et al., 2013). Here we describe how exome sequencing data from related affected subjects was used to initially identify candidate variants in ITGB4. Follow up studies in other samples provide additional support for the involvement of this gene in the aetiology of SCZ and bipolar disorder. Further support comes from data from 872 subjects from the Western Australian Family Study of Schizophrenia and 36,355 controls who reported two SCZ case only *ITGB4* variants (McCarthy et al., 2017). However, it should be noted that for rs147480547 and rs145976111 the MAF (minor allele frequency) observed in both sets of controls is lower than in the Non-Finnish European samples in the ExAC database of aggregated exome sequencing data from large-scale sequencing projects (Lek et al., 2016). Indeed, the ExAC allele frequencies are similar to what we observe in the cases even in the subset of samples that do not include individuals with known psychiatric diagnoses. Thus, the findings for these variants appear to be driven by the fact that they are unexpectedly uncommon in the control samples. By contrast, rs750367954 is very rare and occurs at a frequency of 0.00012 in the non-Finnish European samples in the current version (0.3.1) of ExAC and at a frequency of 0.000025 in the non-psychiatric subjects.

*ITGB4* encodes the integrin beta 4 subunit. Integrins are a large family of heterodimeric receptors for laminin, an extracellular matrix protein which plays a fundamental role in cellular interactions, motility and signalling during development (Tarone et al., 2000). Integrins consist of α and β subunits. The main isoform of the β4 subunit consists of 1882 amino acids. The first 27 amino acids form a signal peptide, the next 683 amino acids form an extracellular domain that is followed by a short transmembrane domain. The C-terminus of the protein is an exceptionally long cytoplasmic domain of 1089 residues. The length of the cytoplasmic domain suggests the importance of the interaction of the β subunit with cytoplasmic proteins (Hogervorst et al., 1990; Suzuki and Naitoh, 1990; Tamura et al., 1990). There is experimental evidence for interaction between the cytoplasmic domain of *ITGB4* with *COL17A1*, *ERBIN*, *BP230*, *BP180*, *DST* and *RAC1* (Favre et al., 2001; Hamill et al., 2009; Hopkinson and Jones, 2000; Koster et al., 2003). Integrin β4 is found primarily in epithelial cells and binds with integrin alpha (α) 6 (*ITGA6*). It is of interest that the *ITGΒ4*/*ITGA6* complex binds to the EGF-like domain in Neuregulin-1 (*NRG1*) (Ieguchi et al., 2010) and variants in the *NRG1* gene have been previously associated with SCZ (Craddock et al., 2005; Mei and Xiong, 2008). All three *ITGB4* variants identified here are located in the long cytoplasmic domain, making them likely to have a functional impact on the interaction of integrin β4 with other cytoplasmic proteins.

Integrin β4 is involved in cell-cell and cell-matrix adhesion. In addition to epithelial cells it is expressed in astrocytes, Schwann cells and neurons (Jaakkola et al., 1993; Milner and Campbell, 2006; Su et al., 2007) and it is abundantly expressed in the developing cerebral cortex (Lv et al., 2008). Integrin β4 is important for the interaction of Schwann cells with the basal lamina and axons, and it could influence the structure of myelinating Schwann cells (Su et al., 2008). Integrin β4 is also believed to be a key factor in neuronal survival and apoptosis signal transduction pathways (Lv et al., 2008; Lv et al., 2006; Su et al., 2007).

We also present *in vitro* evidence from our functional studies to suggest that haplotypes comprising the minor alleles of rs147480547 and rs145976111 lead to an increase in cell proliferation with increased cellular metabolic activity in human neuroblastoma cells transfected with both variant alleles together. Integrin β4 is expressed in human neuronal stem/precursor cells (Flanagan et al., 2006) and downregulation inhibits mouse neural stem cell differentiation (Su et al., 2008). It is well established that the integrin β4 and α6 complex induces c-Src signalling and mTOR activation which is important for stimulating transcription and translation of cancer related genes (Muranen et al., 2017; Soung et al., 2013). Interestingly, the antipsychotic penfluridol has been shown to significantly reduce tumor growth and induce apoptosis in cancer cell lines through inhibition of integrin signalling. This effect was mediated by a decrease in the expression of *ITGΒ4* (Ranjan et al., 2016). Our functional studies demonstrate that *ITGB4* SNP variants rs147480547 and rs145976111 further increase cytoplasmic activity and cell growth in comparison with wild type *ITGB4*. It is thus likely that expression of these variants in *ITGB4* increase cell proliferation by facilitating cell-cell interactions through upregulation of cell signalling. An important limitation of this study is that our functional assays relied on overexpression of ITGB4 in a proliferative cell line. The use of induced pluripotent stem cells derived from patients carrying these variants would likely provide a clearer picture of the functional effects of these variants.

Here we report co-segregation of three rare, protein-changing variants in the *ITGB4* gene with SCZ in two families, with one family presenting a haplotype consisting of two of these variants. We provide further evidence from case control cohorts of subjects that had been exome sequenced or directly genotyped that variants in these families may contribute to susceptibility to SCZ and/or BP in the wider population. We also find support for a role of the *ITGB4* gene in susceptibility to SCZ through a weighted burden test in exome data from a SCZ trio sample and from the literature. Finally, we demonstrate that when these variants are present on the same haplotype there is an increase in cell proliferation *in vitro.* Whilst none of the results obtained provides unequivocal evidence for the role of *ITGB4* in susceptibility to psychotic illness, considered together they are of interest and justify further investigation this gene and its variants in schizophrenia.

The following are the supplementary data related to this article.

Supplementary table 1 and 2. Image 1

Supplementary Table 3 SLPs obtained from weighted burden analysis of cases versus pseudo-controls in Bulgarian trios. Supplementary Table 3

Supplementary Fig. 1 The location of ITGB4 variants identified in the UK10K, the UCL sample and the Bulgarian Trio sample along with protein domains. Supplementary Fig. 1

Supplementary Fig. 2 QQ plot of signed log10 p-values for weighted burden analysis in the Bulgarian trio sample. Supplementary Fig. 2

## Role of the funding source

The funding bodies had no role in the analyses or writing of the manuscript, or the decision to submit this work for publication.

## Contributors

Andrew McQuillin, David Curtis and Nick Bass designed of the study and managed the analysis. Niamh O'Brien and Alessia Fiorentino wrote the first draft of the manuscript and in the opinion of all authors should be considered as joint first authors. Christopher Rayner, Mariam Al Eissa and Chiara Petrosellini contributed with data. Sally Sharp contributed with interpretation of results. All authors contributed to the writing of the manuscript and have approved the final version.
