# Supplementary material for: Rare variant analysis in multiply affected families, association studies and functional analysis suggest a role for the ITGΒ4 gene in schizophrenia and bipolar disorder
Source: Schizophr Res. 2018 Sep;199:181–8. doi: 10.1016/j.schres.2018.03.001 (PMC6179966; doi:10.1016/j.schres.2018.03.001)
Supplement: Supplementary file 1 — Supplementary table 1 and 2. [file mmc1.docx]

| **Name** | **Primer Sequence** |
| --- | --- |
| *ITGB4*_rs147480547_ALG | GAAGGTGACCAAGTTCATGCTGGCTTTGCCACTCATGCCG |
| *ITGB4*_rs147480547_ALA | GAAGGTCGGAGTCAACGGATTCTGGCTTTGCCACTCATGCCA |
| *ITGB4*_rs147480547_C1 | CCCGCCCTCACCCAGCTCT |
| *ITGB4*_rs750367954_ALC | GAAGGTGACCAAGTTCATGCTCAGGGCCAGCCACCGC |
| *ITGB4*_rs750367954_ALT | GAAGGTCGGAGTCAACGGATTCTCAGGGCCAGCCACCGT |
| *ITGB4*_rs750367954_C2 | GCTGTCTCCATCCACCCGGAAT |
| *ITGB4*_rs145976111_ALC | GAAGGTGACCAAGTTCATGCTATGATGGTGATGTTTACCAGGCG |
| *ITGB4*_rs145976111_ALT | GAAGGTCGGAGTCAACGGATTGATGATGGTGATGTTTACCAGGCA |
| *ITGB4*_rs145976111_C1 | GCAGCTGCTGGTGGAGGCCAT |
| *ITGB4*_17_73745042_ALC | GAAGGTGACCAAGTTCATGCTGAGCTGGACGTGGAAACG |
| *ITGB4*_17_73745042_ALT | GAAGGTCGGAGTCAACGGATTGCTGAGCTGGACGTGGAAACA |
| *ITGB4*_17_73745042_C2 | AAGCTCCTGGAGCTGCAAGAAGTT |

Supplementary Table 1. Primers used for genotyping each of the *ITGB4* variants in the UCL cohort.

| Isoforms | rs750367954 | | rs147480547 | | rs145976111 | |
| --- | --- | --- | --- | --- | --- | --- |
|  | SIFT | Polyphen | SIFT | Polyphen | SIFT | Polyphen |
|  | Deleterious | Benign | Deleterious | Benign | Deleterious | Probably Damaging |
| ENST00000200181 | 0.01 | 0.011 | 0.01 | 0.231 | 0 | 0.999 |
| ENST00000339591 | 0.01 | 0 | 0.01 | 0.335 | 0 | 0.998 |
| ENST00000449880 | 0.01 | 0 | 0.01 | 0.335 | 0 | 0.998 |
| ENST00000450894 | 0.01 | 0.004 | 0.01 | 0.006 | 0 | 0.95 |
| ENST00000579662 | 0.01 | 0.004 | 0.01 | 0.006 | 0 | 0.95 |

Supplementary Table 2. Prediction of variant impact on protein structure from Poly-Phen2 and SIFT for all isoforms of ITGΒ4.
