# Supplementary material for: Rare variant analysis in multiply affected families, association studies and functional analysis suggest a role for the ITGΒ4 gene in schizophrenia and bipolar disorder
Source: Schizophr Res. 2018 Sep;199:181–8. doi: 10.1016/j.schres.2018.03.001 (PMC6179966; doi:10.1016/j.schres.2018.03.001)
Supplement: Supplementary Fig. 1 — The location of ITGB4 variants identified in the UK10K, the UCL sample and the Bulgarian Trio sample along with protein domains. [file mmc3.pptx]

## Slide 1
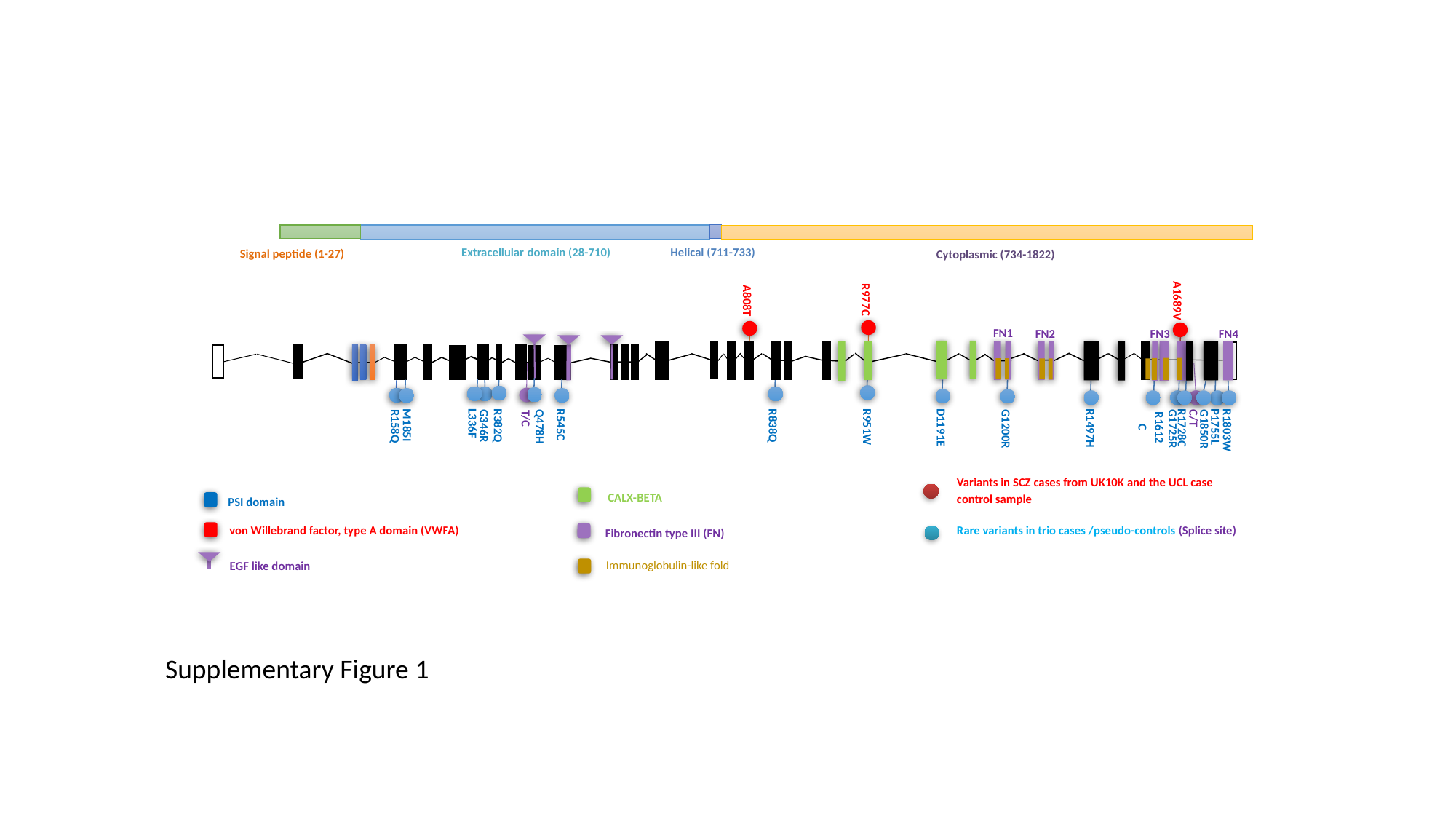

Helical (711-733)
Signal peptide (1-27)
Cytoplasmic (734-1822)
Extracellular domain (28-710)
R977C
A1689V
A808T
FN1
FN3
FN4
FN2
C/T
T/C
R838Q
P1755L
R951W
R1612C
D1191E
R1728C
R1497H
G1200R
G1725R
G1850R
L336F
R1803W
R545C
G346R
Q478H
R382Q
M185I
R158Q
Variants in SCZ cases from UK10K and the UCL case control sample
Rare variants in trio cases /pseudo-controls (Splice site)
CALX-BETA
PSI domain
von Willebrand factor, type A domain (VWFA)
Fibronectin type III (FN)
EGF like domain
Immunoglobulin-like fold
Supplementary Figure 1
