# Supplementary figures and images for: Rare variant analysis in multiply affected families, association studies and functional analysis suggest a role for the ITGΒ4 gene in schizophrenia and bipolar disorder
Source: Schizophr Res. 2018 Sep;199:181–8. doi: 10.1016/j.schres.2018.03.001 (PMC6179966; doi:10.1016/j.schres.2018.03.001)

## Slide 1
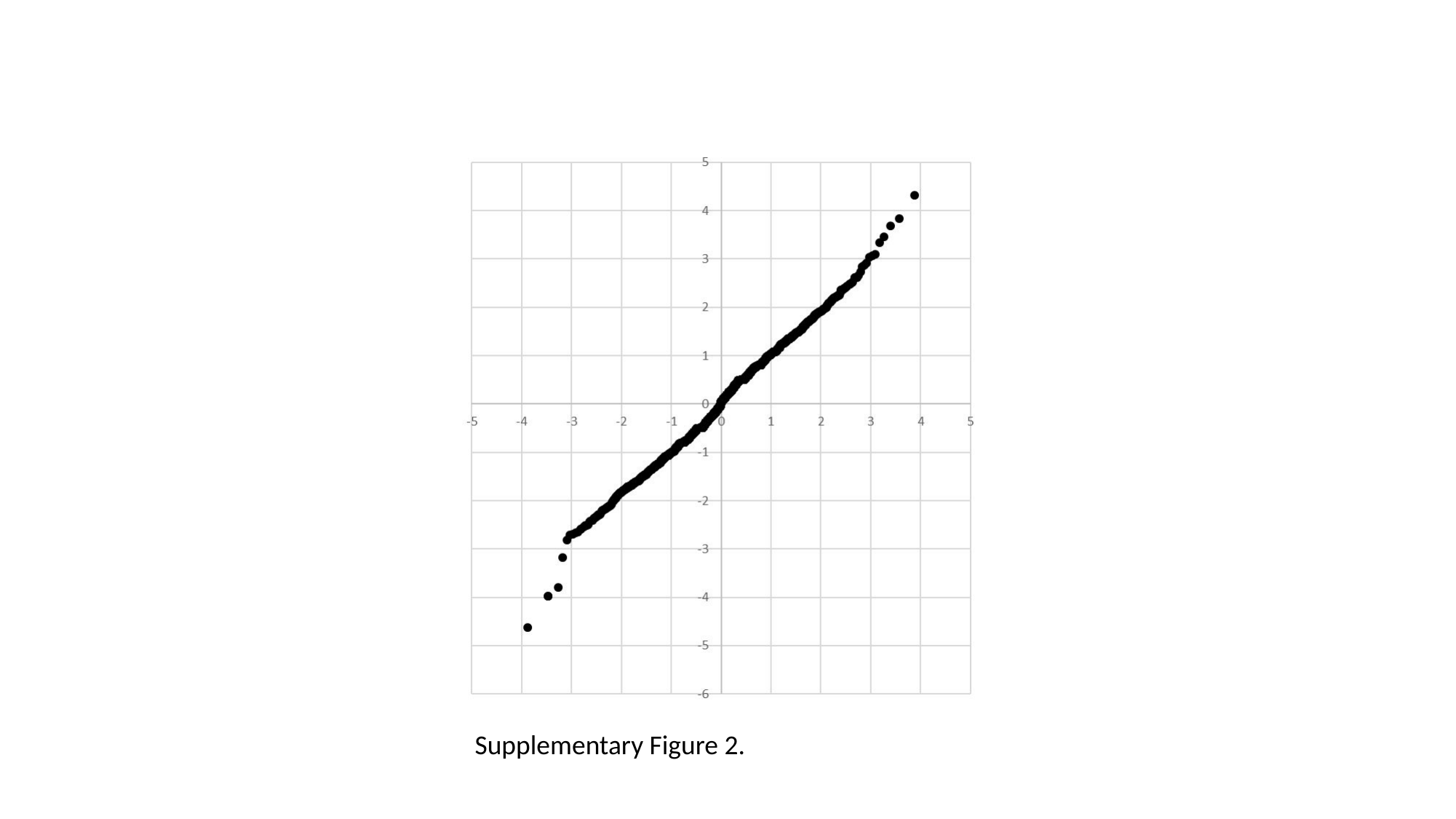

Supplementary Figure 2.

Supplement: Supplementary Fig. 2 — QQ plot of signed log10 p-values for weighted burden analysis in the Bulgarian trio sample. [file mmc4.pptx]
